# Interplaying Factors That Effect Multiple Sclerosis Causation and Sustenance

**DOI:** 10.5402/2012/851541

**Published:** 2012-01-23

**Authors:** Emanuel Calenoff

**Affiliations:** Enteron, Inc., 7030 Lattimore Drive, Dallas, TX 75252, USA

## Abstract

The author hypothesized that multiple sclerosis (MS) is a humoral autoimmune disease, caused by faulty interplay between myelin-specific, dimeric IgE, specifically competing non-IgE antibodies and IgE-triggered degranulating mast cells. The principal fault was believed to be insufficient quantity of protective, specific non-IgE antibodies. Also conjectured was the possibility of an unexpected and adverse immune suppression caused by none-MS pharmaceuticals being consumed by patients for their MS or for other conditions. To test both hypotheses, a mimotopic, peptide antigen-based, serum immunoassay was developed to measure dimer-bound IgE excess among MS patients, wherein the IgE specifically complexes with two or more myelin surface epitopes at an interval of 40–100 Angstroms, a separation critical for mast cell degranulation and cell damaging effect. MS test sensitivity and specificity, when analyzing five previously untreated patients for dimeric IgE presence, was 100%. In direct comparison, twenty age- and gender-matched female and male control subjects were test negative. Analysis of 35 multiple sclerosis patients, who were concomitantly being treated with potentially immunosuppressive pharmaceuticals, appeared to show the substances' negative effect upon MS causation, progression, or specific immunoassay performance. Therefore, MS is likely an autoimmune disease caused by IgE-mediated mast cell degranulation possibly in conjunction with immunosuppressive agents.

## 1. Introduction

While it is commonly understood that multiple sclerosis (MS) is an autoimmune disease of multifactorial etiology, the exact mechanism of causation has not yet been elucidated. Consequently, disease-specific MS therapy has not advanced beyond the use of interferons and immunosuppressive agents, the application of which is now more than thirty-year old [1, 2] and of questionable, long-term efficacy [3].

Based upon his early work [4] and the work of other investigators [5–11], the author hypothesized that MS is a humoral autoimmune disease, caused by faulty interplay between myelin-specific IgE, competing specific non-IgE antibodies, and IgE-triggered, degranulating mast cells. Affected mast cells are likely to expel proteolytic enzymes and possibly other factors which damage or destroy targeted myelin and the axons that are sheathed by it.

To test the hypothesis, a mimotopic, peptide antigen-based, serum immunoassay was developed to measure dimer-bound IgE excess among MS patients, wherein the IgE specifically complexes with two or more myelin surface epitopes at an interval of 40−100 Ångströms, a separation critical for mast cell degranulation [12] and tissue-damaging effect.

Because MS patients often consume numerous medications, some started before observed disease onset, questions arise about the medications' effect upon (1) disease origin, (2) serum antibody test function, and (3) hindrance of normal and protective, humoral immune processes. 

Epitope-specific IgE is but one isotype involved in the myelin inflammatory process as other investigators have also documented the presence of myelin-specific IgA, IgG, and IgM [13]. Concomitantly present, the differing isotypes are likely to be cross-competitive for epitopic antigens. An analytical method was therefore developed to measure this potential competition and a determination made whether the competition exists and is likely part of the MS autoimmune process. 

The analytical method entailed quantification of the ratio of myelin epitope-specific IgE relative to the sum of the matching myelin-specific non-IgE isotypes. In order to simplify the process, the non-IgE antibody level was determined by measuring epitope-specific human kappa plus lambda chains and subtracting the matching epitope-specific IgE. With experience, it became obvious that the specific IgE subtraction was unnecessary as the IgE was exceedingly small in comparison to the matching non-IgE antibodies.

Thereafter, the evolved MS test employed the formula: IgE/(kappa + lambda). Individual humoral epitopes were mimicked by single peptides that were 5 amino acids in length [4], the size that the author had previously estimated would fit into a single antibody Fab site.

When analyzing surface pentameric structures of individual myelin proteins for potentially serving as humoral epitopes (Section 2) and comparing each structure against surface pentamers on all human genome proteins, it was noted that singular, potentially MS-associated, mimotopic peptides displayed amino acid sequences that were located on the surface of a single, specific myelin protein and on no other protein transcribed from the human genome. Those unique pentamers were employed in the MS test development. 

## 2. Materials and Methods

The Hopp and Woods hydrophilicity method for locating epitopic sites on linear protein sequences [15] was used to predict the humoral epitopes on (a) myelin proteolipid protein (PLP, [16]); (b) myelin oligodendrocyte glycoprotein (MOG, [17]); (c) myelin basic protein (MBP) Isoforms 1 [18], 2 [19] and 3 [20]; (d) oligodendrocyte myelin glycoprotein [21], (e) Claudin 11 [22]. 

In order to estimate the functional distance (in Ångströms) between epitopes on the surface of each myelin protein, as depicted on its Hopp and Woods plot, the following tasks were per formed.

1. The average diameter of constituent amino acids was determined by (a) comparing the mass of each amino acid relative to the mass of alanine with its known diameter of 6.9 Ångströms [14]; (b) multiplying individual mass ratios times 6.9 Ångströms to obtain estimated amino acid diameter s for the non-alanine amino acids; (c) averaging the resulting amino acid diameters to obtain a n overall average amino acid diameter of 10.6 Ångströms (Table 1).
2. Individual Hopp and Woods, Excel X-Y plots of each protein's amino acid sequence were modified so as to only depict the protein's hydrophilic surface, either extracellular or intracellular, as if flattened by trimming away all amino acids that were functionally hydrophobic, and likely to be located in the protein interior, but leaving 2 on each hydrophilic edge to reflect infolding toward the protein center (Figures 4, 5, 6, 7, 8, 9, 10, 11, 12, 13, 14, 15).
3. The bridging distance between dimeric surface epitopes on individual myelin proteins was estimated by multiplying the intervening amino acids number by 10.6 Ångströms per amino acid.
4. Because a protein surface is not flat but oscillates in depth, sera from an age- and gender-varied MS-negative control group were sequentially tested to measure dimer-point, myelin epitope-specific IgE/(kappa + lambda) values while reducing the estimated dimeric distances in 5 percent intervals to find a reduction percentage to use as a factorial adjustment which simulated staircase dips in normally rolling surface contours of proteins and thus afforded functionally negative test results for the controls (no ideal dimers present). A 25% reduction of the linear distances attained in Step 3 between dimeric epitopes afforded test-negative results for all initially tested, negative-control serum samples by exhibiting no more than one dimer point as being IgE/(kappa + lambda) test positive.
5. In validation, when testing positive control MS patient sera and employing the 25% reduction factor, dimer bridging, test-positive values were attained whose corresponding epitopes' separation ranged from between 40 to 100 Ångströms, thus indicating a likelihood of mast cell degranulation. Claudin 11 and oligodendrocyte myelin glycoprotein lacked IgE dimer bridging sites and were, therefore, not analyzed further. Pathological dimer bridging values and locations are depicted in Figures 4–9 for MOG and Figures 10–15 for MBP.

**Figure 1:**
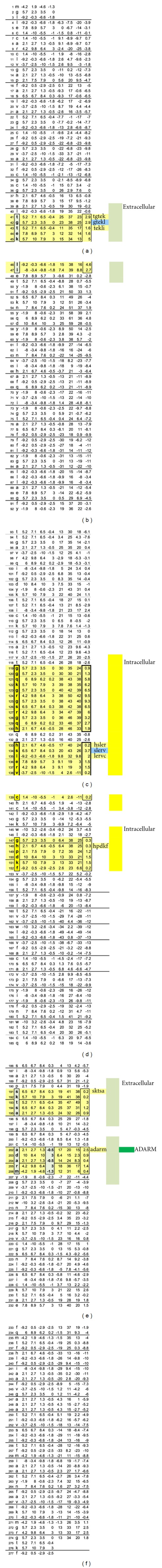
Check description in Section 2.

**Figure 2:**
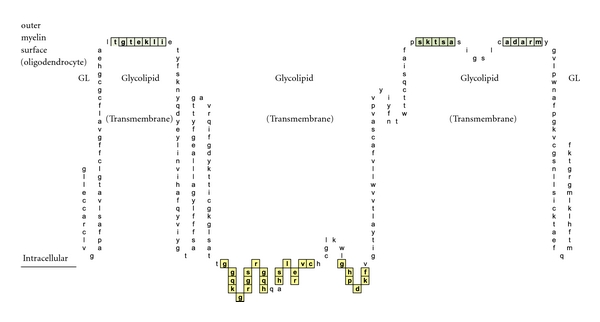
A schematic illustration of myelin proteolipid protein Isoform 1 is shown. Depicted are: (a) amino acid sequence portions that are net hydrophilic and located on the myelin protein (oligodendrocyte) surface (blue-green highlight); (b) portions that are net hydrophobic and project inwardly within the myelin glycolipid layer (uncolored); and (c) portions that are hydrophilic and intracellular (yellow highlighted).

**Figure 3:**
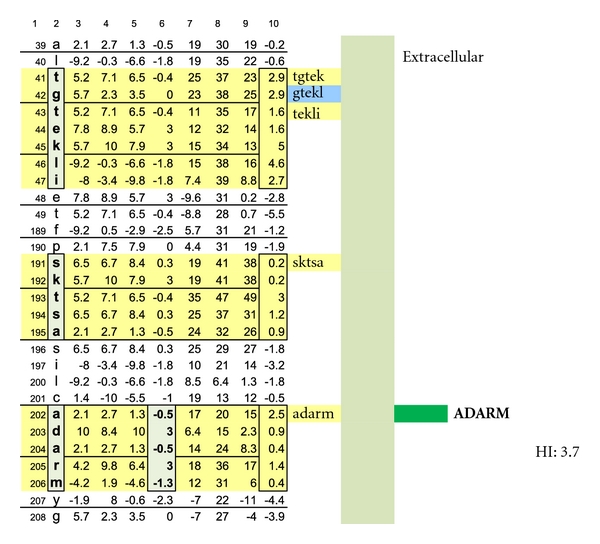
A schematic drawing is shown wherein the location of a myelin-specific epitope, ADARM, is illustrated by performing a pictorially functional readjustment of Figure 1(a), removing rows 1–38, 48-49, 50–116, 155–188, 198-199, and 209–277 to visualize the 3 hydrophilic surface platforms, susceptible to autoantibody binding. The individual platforms encompass amino acids number 39−49, 189–197, and 200–208. Two intracellular, hydrophilic platforms encompass amino acids number, 117–141 and 145–154. The unique, corresponding amino acid hydrophilic indices, −0.5, 3, −0.5, 3, and −1.3 depicted in column 6.

A similar contour-mapping approach was not used for PLP because it has but one structurally unique epitope expressed on the myelin surface, ADARM (Figures 1, 2, 3). However, PLP molecules are highly prevalent on myelin and correctly spaced (65–71 Ångströms) between monomers [23] so as to serve as ideal IgE dimer-binding sites, notwithstanding allowance for the 25% rolling contour adjustment. 

In Figure 1, Proteolipid protein Isoform 1 (PLP1) is displayed as a Hopp and Woods XY plot with eleven vertical columns. The leftmost column depicts the amino acid sequence number of the protein chain. The second column from the left lists the sequential, corresponding amino acids. The sixth, left-most, column displays the hydrophilic index (HI) of each listed amino acid. The tenth column from the left depicts the sum-of-seven, continuous amino acids, hydrophilic index value of each amino acid derived by adding to its hydrophilic index (HI) the indices of the 3 amino acids that precede it plus the indices of the three amino acids that follow it. Areas that are net hydrophilic (yellow highlighted) are apt to be on the protein surface while those that are net hydrophobic (uncolored) would be on the protein edge or imbedded within the protein center. The protein surface can either be extracellular or intracellular.

**Figure 4:**
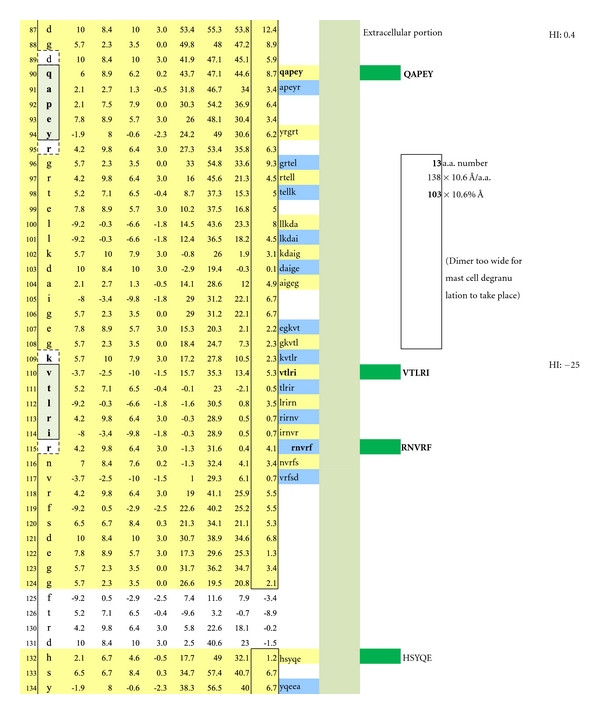
Displayed is the measured distance between two IgE autoantibodies if each was to bind a potential epitopic dimer site (QAPEY and VTLRI) with each site incorporating five, uniquely sequenced, contiguous amino acids flanked on either end by a nonreactive, normally present amino acid thus making a 7 amino acid, antibody binding footprint. Each intervening amino acid between epitopes is estimated to be 10.6 Ångströms in width. When the interfootprint dimer distance analysis is performed, the potential dimer between QAPEY and VTLRI is inadequate for mast cell degranulation because there are 13 intervening amino acids between the two epitopes, and this is equivalent to a distance of 103 Ångströms, which is 3 Ångströms above the mandated upper limit of 100 Ångströms. HI: peptide hydrophilic index.

**Figure 5:**
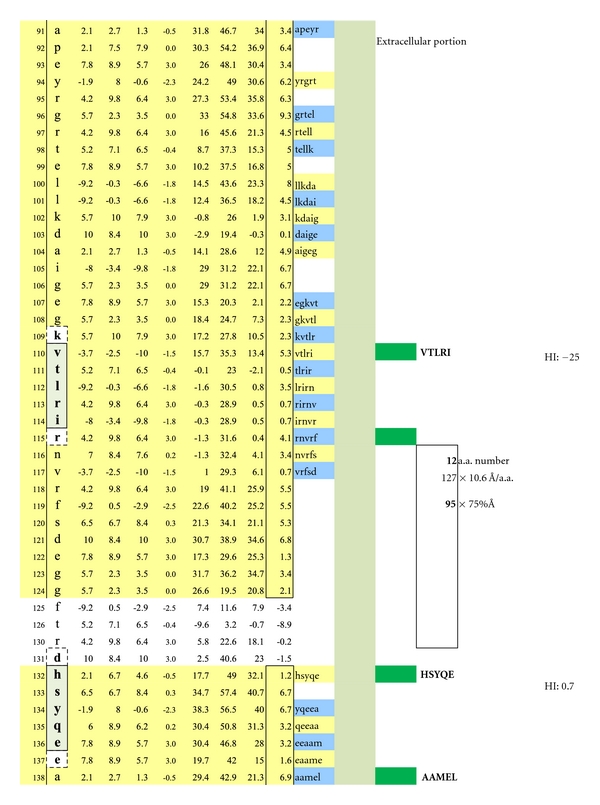
A potentially functional dimer site with an interval distance of 95 Ångströms between the epitopes VTLRI and HSYQE is illustrated.

**Figure 6:**
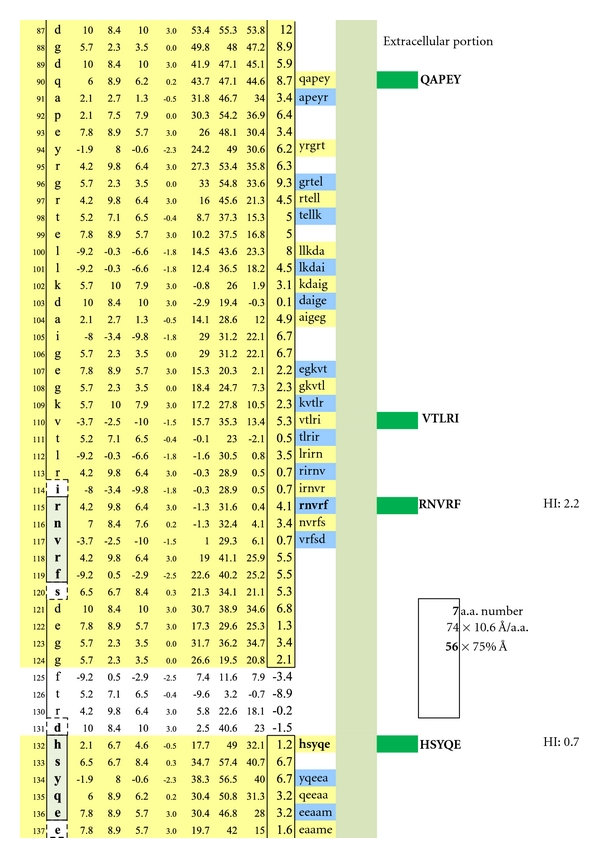
A potentially functional dimer site with an interval distance of 56 Ångströms between the epitopes RNVRF and HSYQE is illustrated.

**Figure 7:**
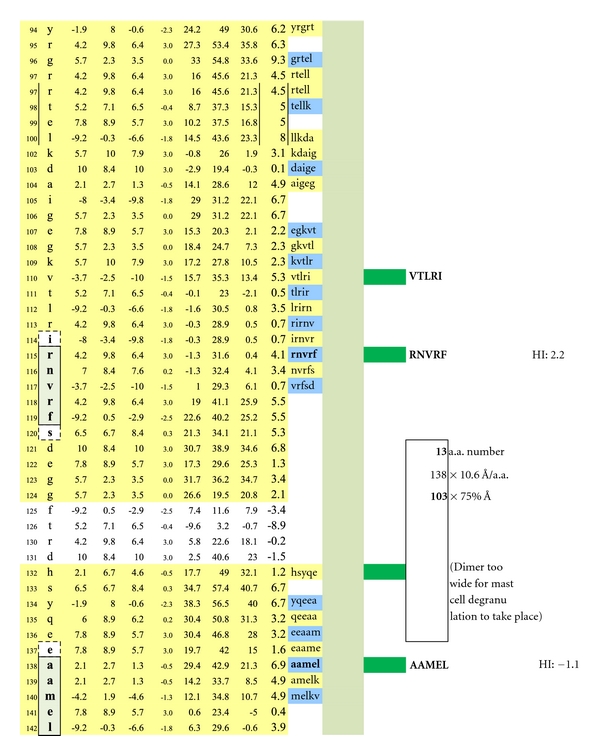
A potentially non-functional dimer site with an interval distance of 103 Ångströms between the epitopes RNVRF and AAMEL is illustrated.

**Figure 8:**
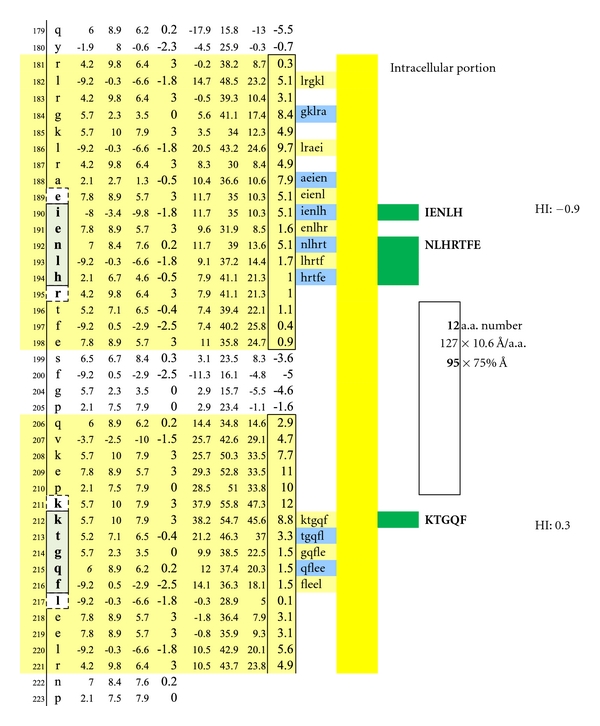
A potentially functional dimer site with an interval distance of 95 Ångströms between the intracellular MOG epitopes IENLH and KTGQF is illustrated. The epitopes' complexing with specific IgE antibodies and mast cells likely hinges upon disruption of the overhanging oligodendrocyte membrane surface.

**Figure 9:**
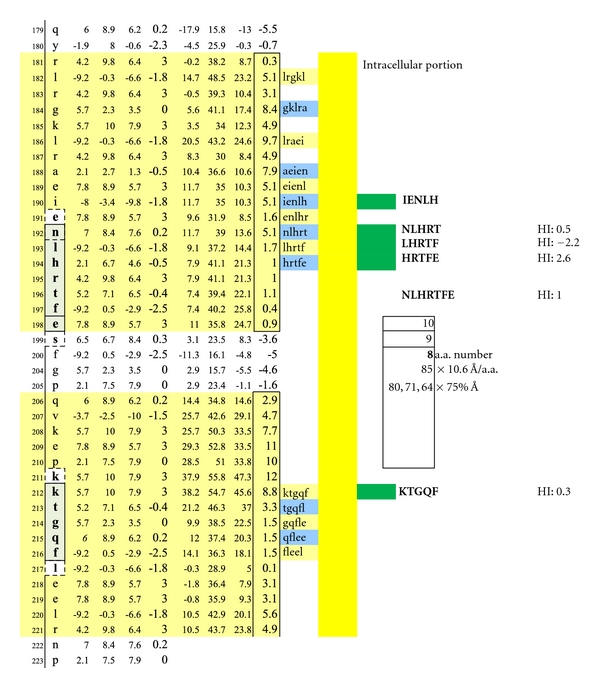
Illustrated are three potentially functional dimer sites with interval distances of 80, 71, and 64 Ångströms between the interlayer MOG epitopes NLHRT and KTGQF, LHRTF and KTGQF, and HRTFE and KTGQF. Dimeric IgE complexing hinges upon disruption of the overhanging oligodendrocyte membrane surface and facilitated intracellular antibody inflow. For serum antibody immunoassay purposes, the longer, inclusive peptide NLHRTFE can be used together with KTGQF as both peptides are sufficiently hydrophilic when coupled with the peptide-solubilizing, amino-ADOOA-ADOOA linker.

MOG has been shown to be differentially expressed in various isoforms. However, for the purpose of identifying the potential array of MOG humoral epitopes possible on all isoforms and the epitopes' utility in dimer formation, analysis of MOG Sanger Institute Isomer 1 (Figures 4–9) proved sufficient. The analysis illustrated the presence of two potential disease-functional dimer sites on the oligodendrocyte, extracellular MOG portion (Figures 5 and 6) and two potential subsurface, intracellular dimer sites (Figures 8 and 9) if the latter were somehow immunosurveillance exposed by myelin surface (oligodendrocyte surface) disruption. By experience, accurate representation of an antibody binding site mandated exhibiting a unique pentamer flanked by an amino acid on each side. 

**Figure 10:**
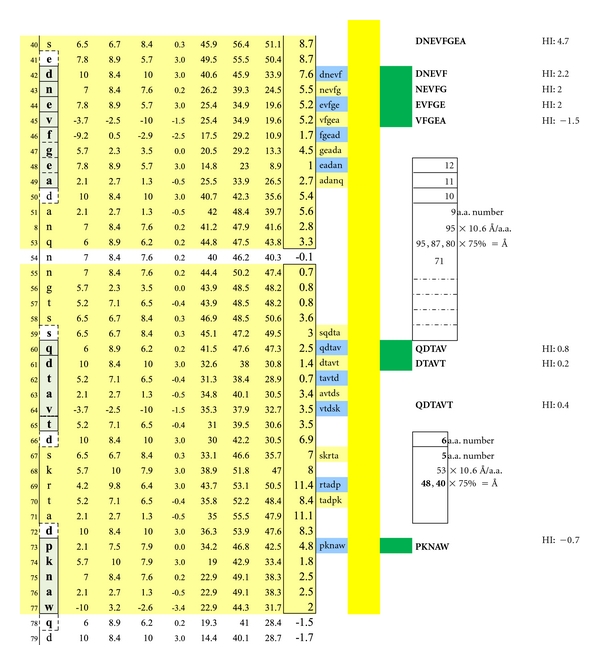
Seven structurally unique epitopes located on the outer surface of myelin basic protein (MBP) Isoform 1 are illustrated. The dimer group 1 combinations encompass the overlapping, epitopic pentamers DNEVF, NEVFG, EVFGE, and VFGEA coupled to QDTAV. The dimer group 2 combinations encompass the pentamers DNEVF, NEVFG, EVFGE, and VFGEA coupled to DTAVT. The dimer group 3 combinations encompass the overlapping, epitopic pentamers QDTAV and DTAVT individually coupled to PKNAW. Dimer group 1 displays epitope intervals that are 95, 87, 80, and 72 Ångströms. Dimer group 2 displays epitope intervals that are 88, 80, 72, and 64 Ångströms. Dimer group 3 displays epitope intervals that are 48 and 40 Ångströms. Dimeric IgE complexing hinges upon disruption of the overhanging oligodendrocyte membrane surface and facilitated intracellular autoantibody inflow. For serum antibody immunoassay purposes, the longer, inclusive peptide DNEVFGEA can be used together with QDTAVT and QDTAVT used together with PKNAW as all three peptides are sufficiently hydrophilic when coupled with the peptide-solubilizing construct, amino-ADOOA-ADOOA linker.

**Figure 11:**
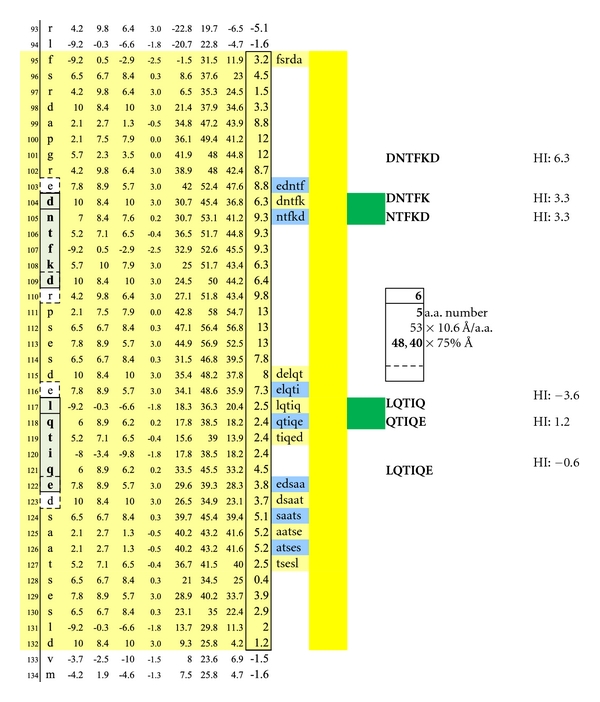
A second set of potentially functional intracellular dimer sites on are displayed on MBP Isoform 1. The inclusive epitope pairs are: DNTFK, LQTIQ and DNTFK, QTIQE and NTFKD, LQTIQ and NTFKD, QTIQE with respective interval distances of 40 and 48 Ångströms. Dimeric IgE complexing hinges upon disruption of the overhanging oligodendrocyte membrane surface and facilitated intracellular autoantibody inflow. For serum antibody immunoassay purposes, the longer, inclusive peptide DNTFKD can be used together with LQTIQE as both peptides are sufficiently hydrophilic when coupled with the peptide-solubilizing, amino-ADOOA-ADOOA linker.

**Figure 12:**
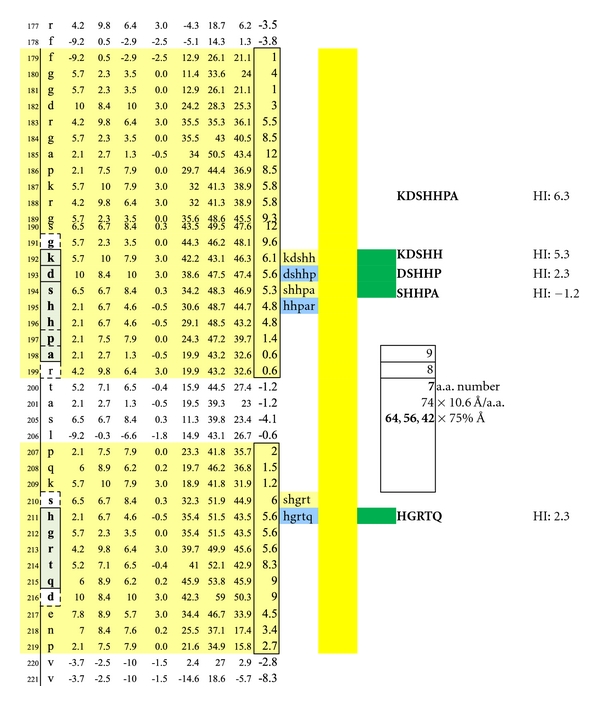
A third set of potentially functional intracellular dimer sites on MBP Isoform 1 is displayed incorporating the epitopes KDSHH, DSHHP, and SHHPA individually coupled to HGRTQ. The dimer epitopes' complexing with specific IgE antibodies likely hinges upon disruption of the overhanging myelin surface and specific antibody inflow. For serum antibody immunoassay purposes, the longer, inclusive peptide KDSHHPA can be used together with HGRTQ as both solubilize readily with the amino-ADOOA-ADOOA linker.

**Figure 13:**
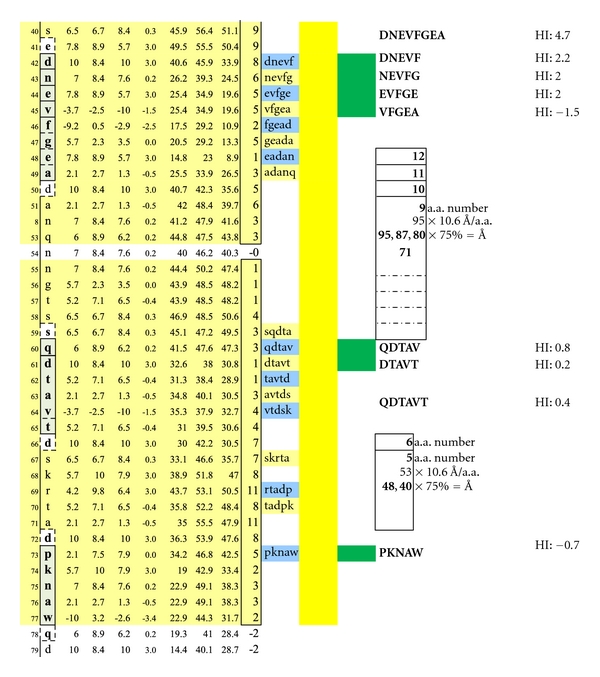
Potentially functional dimer sites on MBP Isoform 2 and conditions match the dimersets on MBP Isoform 1 displayed in Figure 10.

**Figure 14:**
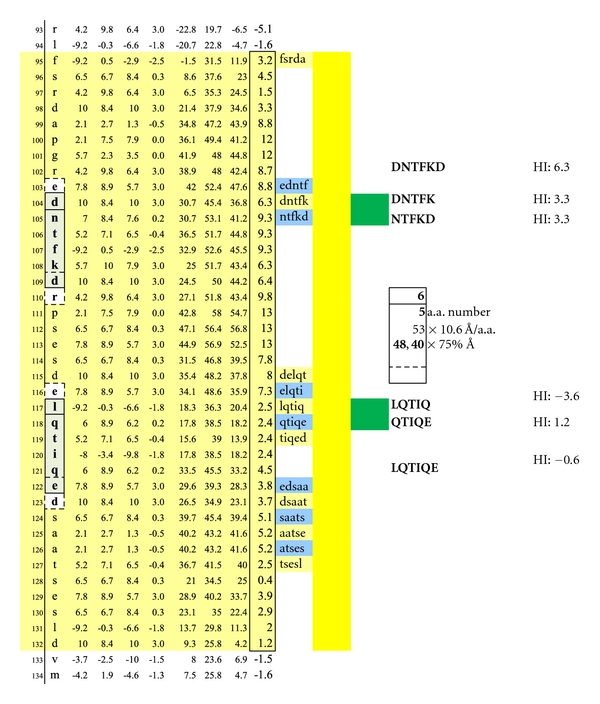
Potentially functional dimer sites and conditions on MBP Isoform 2 match a dimer set on MBP Isoform 1 as displayed in Figure 11.

**Figure 15:**
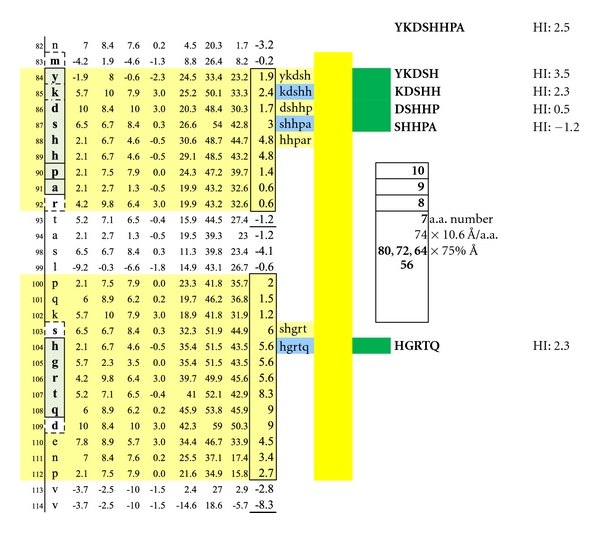
Potentially functional intracellular dimer sites and conditions on MBP Isoform 3 which are similar to the third dimer set on MBP Isoform 1 as displayed in Figure 12.

Myelin basic protein, an oligodendrocyte intracellular protein also potentially accessible by myelin surface disruption, exhibits a more complex array of potentially tar getable epitopes and dimer conditions than PLP or MOG. All potential dimer sites are exhibited by a combination of myelin basic protein Isoform 1 (Figures 10–12), Isoform 2 (Figures 13-14), and Isoform 3 (Figure 15). For epitope-mapping, singularly unique protein surface regions longer than 5 amino acids were subdivided into overlapping pentamers to represent all possible-single-antibody binding sites (i.e., Figure 10). 

### 2.1. Microtiter Test Plate Layout

**Figure 16:**
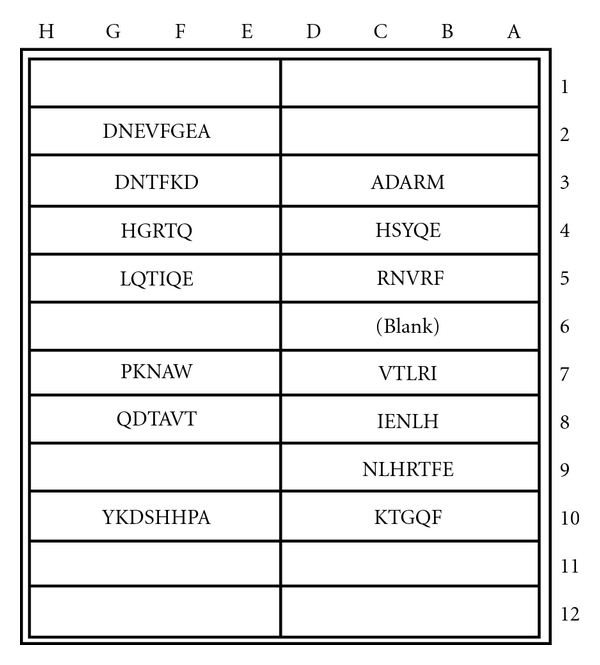
MS test microplate layout of mimotopic peptide antigens covalently coupled to individual test wells. The test plate format was employed for both specific IgE and non-IgE serum antibody determinations.

As depicted in Figure 16, the basic MS test plate contained 14 mimotopic peptides corresponding to the myelin epitopes illustrated in Figures 1 through 15. Each peptide construct (Mimotopes Pty, Clayton, Australia) applied to the microplate wells comprised a mimotopic peptide preceded by an aminated hydrophilic linker, 8-amino-3,6-dioxaoctanoic acid_2_ (amino-ADOOA-ADOOA). The epsilon amino group on peptide lysine residues was blocked with a (4,4-dimethyl-2,6-dioxocyclohex-1-ylidene)-3-methylbutyl (ivDde) group, to prevent undesirable, lateral binding when attaching peptide constructs to test plate wells via their free amide groups.

### 2.2. Peptide Constructs Formulation

Each construct was dissolved in pH 7.2 phosphate buffered saline (PBS) immobilization buffer (Product no. 28372, Thermo Scientific, Rockford, IL, USA), at an assay optimum concentration (Table 2).

### 2.3. Peptide Constructs Application

100 *μ*L of each peptide construct solution was applied in quadruplicate to 96-well, amide-binding, maleic anhydride-activated, white 96- well plates (Thermo Scientific, Product no. 15108). Four wells were left blank for plate background determination. The plates were covered with acetate plate sealers (Thermo Scientific, Product no. 3501) and the construct solutions incubated at 21−26 degrees C for 18−24 hrs.

### 2.4. Plate Blocking Procedure

120 *μ*L of HSA background blocking solution (10 mg recombinant human serum albumin per mL immobilization buffer) was applied per well. The construct/blocking solutions were incubated at 21–26 degrees C for 18–24 hrs and plates aspirated and dried.

### 2.5. Lysine Deprotection Procedure

200 *μ*L of a 2% solution of hydrazine monohydrate (Sigma Chemical Company, St. Louis, MO, Product no. 207942) in DMSO (dimethyl sulfoxide, Thermo Scientific Product No. 20688) was applied per well and incubated at 21–26 degrees C for 10 minutes. The hydrazine solution was aspirated and the procedure repeated two additional times. 250 *μ*L of phosphate buffered saline with 0.05% Tween-20 (PBST, Thermo Scientific Product no. 28320) was applied per well. Plates were incubated at 21–26 degrees C for 30 minutes, aspirated, washed three additional times, aspirated, and dried.

### 2.6. Microplate Storage

After drying at 21−26 degrees C for 18−24 hrs in a clean, covered container, test plates were sealed with acetate plate sealers and stored at room temperature until needed. Individual plates were used for both specific IgE and specific (kappa + lambda) assays.

The *specific IgE immunoassay* entailed use of 100 uL/well of neat subject serum that had been spiked with 1 mg/mL aminated hydrophilic linker solution to neutralize potential, antilinker antibodies (50 *μ*L linker solution per 12 mL serum). Plates were sealed and incubated for 2 hours at 21–26 degrees C and then washed four times with PBST (250 *μ*L/well followed by immediate aspiration). 100 *μ*L of 4 *μ*g/mL biotinylated, goat anti-human IgE was applied per well. (The anti-IgE solution comprised 96 *μ*L of Vector Labs, Burlingame, CA, Product no. BA-3040 added to 11.9 mL of conjugate diluent (10 mg/mL recombinant HSA in PBST +0.25% PEG 4000)). After incubation for 2 hours at 21–26 degrees C, the plates were washed and 100 uL/well of 64 ng/mL streptavidin horseradish peroxidase (Thermo Scientific Product No. 21126 diluted in HSA conjugate diluent) applied. Test plates were incubated for 30 minutes at 21−26 degrees C and then washed. 100 uL/well of Thermo Scientific Chemiluminescence substrate (Product No. 37074) was applied and the plate(s) read 1−3 minut**e**s after application using a Luminoskan Ascent Microplate Luminometer (Thermo Scientific, Waltham, MA, USA).

### 2.7. Specific (Kappa + Lambda) Test Portion

The linker-spiked test serum sample used in the specific IgE assay was diluted 1/25,000 by (a) making a 1/100 dilution mixing together 100 *μ*L linker-spiked serum and 9.9 mL PBST and (b) spiking 11.950 HSA conjugate diluent with 48 *μ*L of the 1/100 diluted serum. Plates were filled with 100 *μ*L/well of 1/25 k diluted serum, sealed, and incubated for 2 hours at 21–26 degrees C. Equal volumes of Vector biotinylated, goat anti-human kappa antibody (BA-3060) plus biotinylated, goat anti-human lambda antibody (BA-3070) were mixed together to form a 50 : 50 biotinylated anti-(kappa + lambda) concentrate (500 *μ*g/mL). 96 *μ*L of the anti-(kappa + lambda) concentrate was mixed with 11.9 mL of HSA conjugate diluent and 100 *μ*L of the resulting solution applied per well. After 2-hour incubation at 21–26 degrees C, plates were washed and 100 uL per well of 16 ng/mL streptavidin horseradish peroxidase solution applied. Test plates were incubated at 21–26 degrees C for 30 minutes, aspirated, and washed. 100 uL/well of Thermo Scientific chemiluminescence substrate was applied and the plates read at 1–3 minutes after application.

### 2.8. Individual IgE/(Kappa + Lambda) Determinations

Specific IgE and matching specific (kappa + lambda) signals were obtained by reading corresponding, matched test plates on the microplate luminometer. Average test values corresponding to individual mimotopic peptides were determined by discarding the highest and lowest of four values and averaging the remaining two. The same procedure was followed for the four background well values, together with calculation of twice the standard deviation of the two-point average. The blank well background was deemed to be its average value plus twice the standard deviation. The plate blank well value was subtracted from each peptide-coated well average to yield a net signal. Kappa + lambda values were multiplied by 25,000 in order to delineate the corresponding neat serum (undiluted) epitope-specific kappa + lambda antibody levels. IgE/(K+L) values were multiplied by 1,000,000 in order to bring each quotient to a positive number if attainable. Test results with negative values or values of less than 0.5 were assumed to be test negative. 

### 2.9. Serum Sample Selection

Disease-positive and disease-negative (control) sera were purchased from BioServe (Beltsville, MD, USA). Multiple sclerosis-positive samples were from patients who had not yet received MS-specific therapy, patients who were taking interferon and/or Copaxone (glatiramer acetate) with or without medication shown to be immunosuppressive (Tables 3(a)–3(e)), and patients who were only being treated with immunosuppressive substances.

## 3. Results

### 3.1. Control Subjects

As depicted in Figures 17 and 18, neither female (*n* = 10) nor male (*n* = 10) age-varied, control serum samples were MS test positive by exhibiting IgE/(kappa + lambda) dimer-positive results. Being positive, the control sera would have been IgE/(kappa + lambda) positive when tested against the PLP ADARM peptide antigen (Figures 1–3) and/or the non-PLP peptides listed on the left-most column of Table 4 that were properly dimer paired. The proper pairings are depicted on Figures 5-6 and Figures 8–15.

### 3.2. Untreated MS Patients

**Figure 19:**
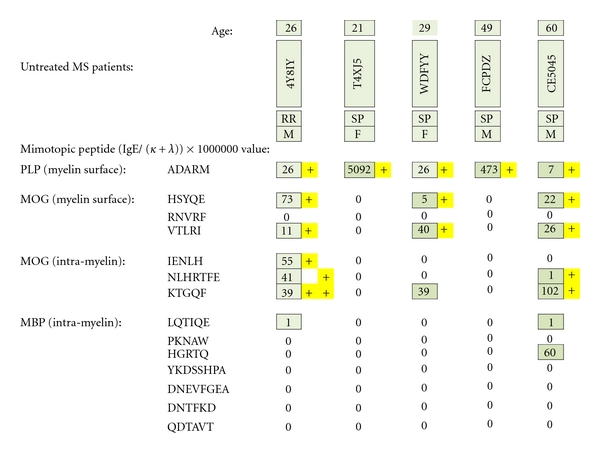
MS test results for serum samples obtained from multiple sclerosis patients (4 Caucasians and 1 African American) who had not yet received pharmacotherapy. Individual epitope- positive results are highlighted with yellow plus signs. To be dimer test positive, MS patients had to be ADARM-specific, IgE/(kappa + lambda)-positive and/or IgE/(kappa + lambda)-positive for the dimer pairs HSYQE and VTLRI, HSYQE and RNVRF, IENLH and KTGQF, and/or NLHRTFE and KTGQF.

Five MS patients who had not taken Copaxone, beta interferons, or immunosuppressive agents were MS test positive (Figure 19). All were IgE dimer positive against PLP. Three of five patients were also MOG dimer-positive. Of the latter group, all three were myelin surface positive and two patients were also positive against subsurface MOG epitopes (positives are highlighted in yellow). None were MBP dimer positive.

### 3.3. Patients Taking Copaxone or Interferon Only (Figure 20)

All three patients were dimer positive against the sole PLP epitope, ADARM, irrespective of whether each had relapsing remitting (RR), secondary progressive (SP), or primary progressive (PP) MS. Individual test scores were lower than scores for the untreated MS group. None was IgE dimer positive against MOG or MBP.

### 3.4. Patients Taking Interferon + Immunosuppressive Agents (Figure 21)

One of twelve patients was PLP dimer test positive. None were positive against MOG or MBP. (Potentially immunosuppressive, ancillary agents are numerically identified in yellow at the bottom of the figure and referenced in Table 3.)

### 3.5. Patients Only Taking Immunosuppressive Agents (Figure 22)

Four of fourteen test subjects were PLP IgE dimer test positive, irrespective of disease classification. None was IgE dimer positive against MOG or MBP. (Immunosuppressive agents are identified in yellow at the bottom of the figure and referenced in Table 3.)

### 3.6. Patients Taking Copaxone plus Immunosuppressive Agents (Figure 23)

Only one of six patients was PLP and MOG dimer test positive. None was MBP dimer positive. (Immunosuppressive agents are identified in yellow at the bottom of the figure and referenced in Table 3.)

## 4. Discussion

The test data is both an affirmation and a significant challenge to the theories and work of other investigators. It affirms the premise that MS is a humoral autoimmune disease as validated by clinicians who have worked with Natalizumab (Tysabri), a humanized monoclonal antibody whose therapeutic use results in a systemic increase in B lymphocytes and, ultimately, plasma cells and specific serum autoantibodies [52–54]. Where it differs is in affording a precise explanation of the multilayered humoral MS process and how it might be remedied.

We have concluded that multiple sclerosis is a complex, humoral autoimmune disease caused by IgE dimer formation on the surface or immediate subsurface of CNS myelin that results in focal mast cell degranulation. The degranulating mast cells likely release proteolytic enzymes and possibly other factors that damage or destroy proximal neurons.

Test results of MS patients taking medication shown to have an immunosuppressive effect suggest either interference with the IgE/competing antibody pathological process or with the MS test itself. This was inferred by the quantitative difference in MS test scores between the patients who were taking no medication and exhibited relatively high test scores, patients who were being treated with a single MS-specific pharmaceutical (beta interferon or Copaxone), but had relatively low positive test scores, and patients who were taking the described immunosuppressive agents, with or without interferon or Copaxone, and were only sporadically MS test positive.

This suggests that the conventional, multiple therapy approach may potentially hinder therapeutic efficacy by altering normal immune function.

**Figure 20:**
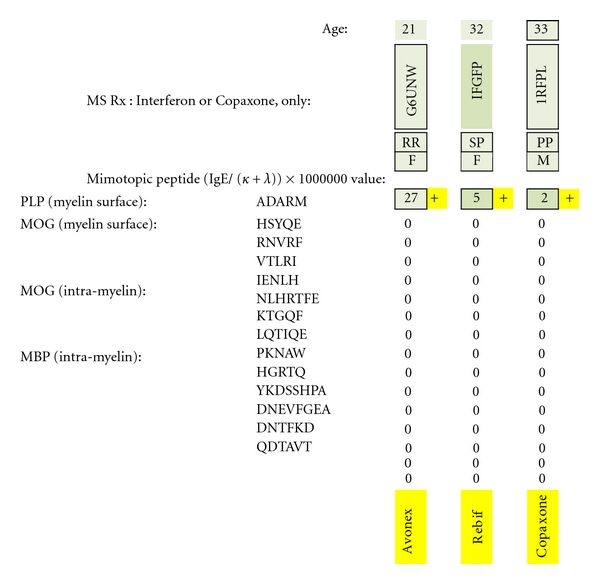
MS test results for serum samples obtained from multiple sclerosis patients only treated with interferon or Copaxone. Individual epitope-positive results (all against the PLP epitope ADARM) are highlighted with a yellow plus sign. Being test-positive to PLP indicates dimer-positive presence because of the PLP monomers' high myelin surface prevalence and adequate intermolecular monomer-to-monomer separation (65–71 Ångströms).

**Figure 21:**
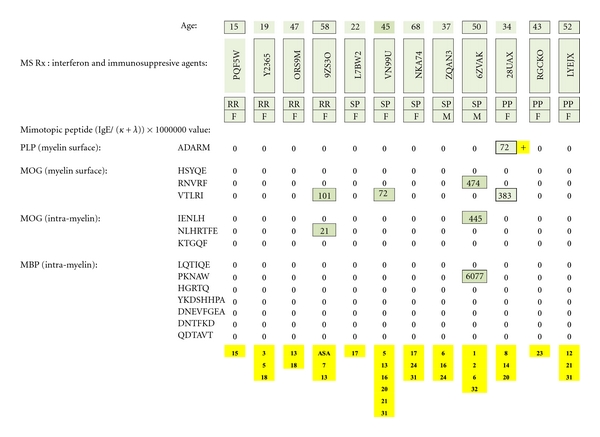
MS test results for serum samples obtained from multiple sclerosis patients treated with interferon plus psychotropic pharmaceuticals and/or other potentially immunosuppressive agents. Individual dimer-positive results (just one) are highlighted with a yellow plus sign. The immunosuppressive substances are identified by yellow-highlighted numbers at the bottom of columnar, individual patient test results and referenced in literary citations provided in Table 3 that are listed at the end of the paper.

**Figure 22:**
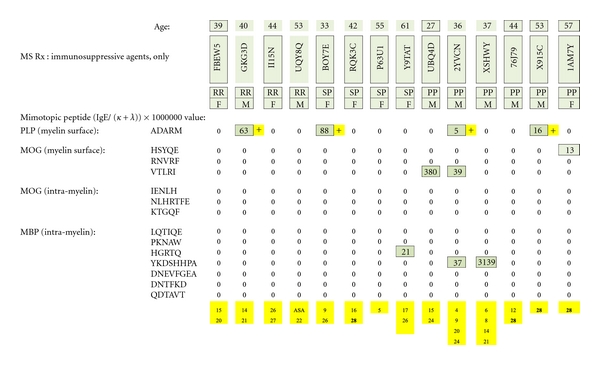
MS test results for serum samples obtained from multiple sclerosis patients only treated with psychotropic pharmaceuticals or other immunosuppressive agents. Individual epitope-positive test results (4 ADARM positives) are highlighted with a yellow plus sign. The immunosuppressive substances are identified by yellow-highlighted numbers at the bottom of the columnar, individual patient test results and referenced in literary citations provided in Table 3 that are listed at the end of the paper.

**Figure 23:**
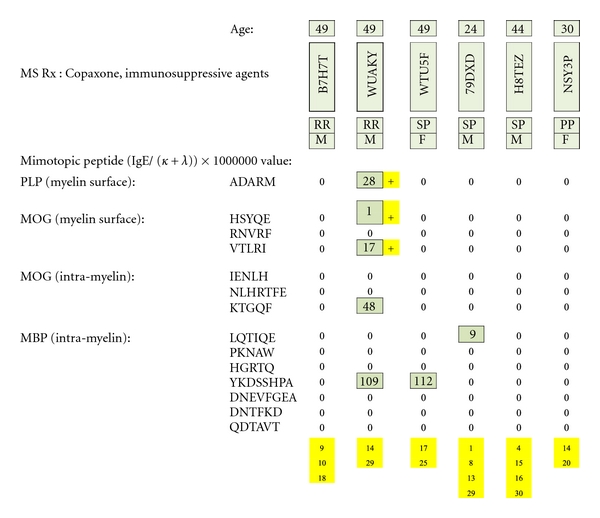
MS test results for serum samples obtained from multiple sclerosis patients treated with Copaxone plus psychotropic pharmaceuticals or other immunosuppressive agents. Individual epitope-positive results (just one tested individual) are highlighted with yellow plus signs. The immunosuppressive substances are identified by yellow-highlighted numbers at the bottom of columnar, individual patient test results and referenced in literary citations provided in Table 3 that are listed at the end of the paper.

As interferon and Copaxone are prescribed because of their MS-specific, immunosuppressive, effect and MS is likely an autoimmune disease, it is reasonable to expect some decrease in humoral immune function among specifically treated patients, as reflected by the low anti-PLP test scores exhibited in Figure 20. However, the test scores of MS patients who were also receiving ancillary substances that have been shown to be immunosuppressive (Figures 21–23), infer that augmentation of immune suppression may not always be therapeutically beneficial. Another possibility is that the MS-specific and ancillary pharmaceuticals may also hinder serum immunoassay function making some or all specific immunoassays diagnostically unreliable.

Also suggested is the possibility that (1) the ancillary medications, having only been in use since the early twentieth century, may have historically played an instigating or promoting role in MS causation and/or progression through disruption of homeostatic, immune system controls.

The absence of MS test-positive results against MBP dimers may have been due to their deep, intracellular location and relative freedom from immune surveillance and pathological action. It may also indicate that (a) successful MBP humoral targeting first calls for myelin surface disruption via action against PLP and/or outer MOG in order to provide penetrating access for anti-MBP antibodies and/or lymphocytes; (b) MBP is an autoimmune target, but of lesser frequency or importance than the epitopes on the outer surface or immediate sub-surface of myelin.

If MS proves to be an IgE dimer-driven, humoral autoimmune disease, as is suggested by the test data, treatment with mimotopic peptides homologous to those used in the diagnostic immunoassay might prove to be therapeutically beneficial by neutralizing anti-myelin IgE antibodies. The therapeutic peptides would need to be administered in such a way as to insure intravascular delivery of quantities sufficient to neutralize all epitope-specific dimeric IgE autoantibodies through antibody-to-peptide complexing or by neutralizing key epitope-specific IgE antibodies whose target epitopes are cornerstones of a number of pathological dimers. The elimination of one or two dimer-point-specific autoantibodies could prevent multi-site-initiated mast cell degranulation and halt the pathological autoimmune process.

Cornerstone dimer-blocking peptides are highlighted in bold within the full array of potentially therapeutic peptides listed in the sixth column from the left of Table 4 and whose corresponding epitopes and dimers are illustrated in Figures 4 thru 15.

To be effective, therapeutic peptides would need to (a) possess an exact structural match with the specific myelin protein epitope; (b) be of sufficient length (5-6 amino acids) to easily fit and be avidly bound by a single autoantibody; (c) be relatively hydrophilic so as to be functionally soluble when injected or ingested; (d) if ingested, be aided in enteric absorption by pharmaceutical agents such as medium-chain fatty acid constructs [55], superporous hydrogels [56], or N-trimethyl chitosan chloride [57]. 

## 5. Summary

Evidence is presented that multiple sclerosis is a humoral autoimmune disease caused by IgE dimer formation on the surface or immediate subsurface of CNS myelin that results in focal mast cell degranulation with release of tissue-damaging enzymes and/or other substances.

## Figures and Tables

**Figure 17:**
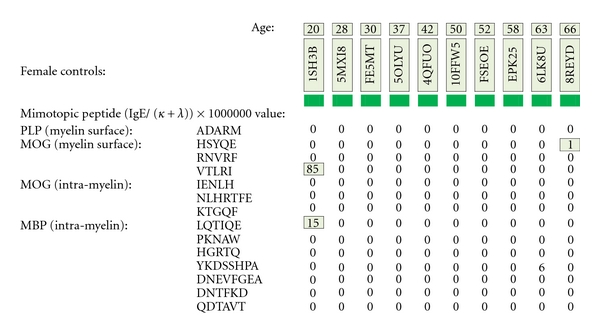
MS test results for ten female control serum samples, ages 20–66, are displayed. Tested samples were from Caucasian and African-American donors who did not have multiple sclerosis. Specific IgE/(kappa + lambda)-positive results are confined to single, nondimer participating epitope.

**Figure 18:**
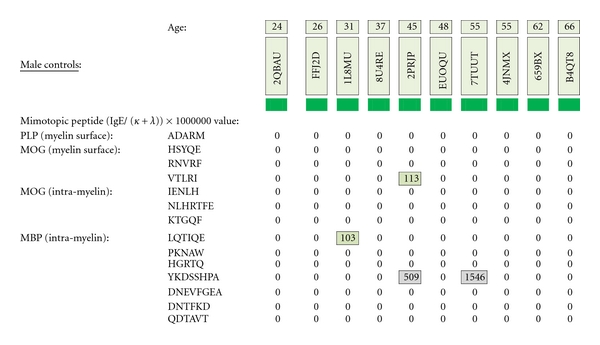
MS test results for ten male control serum samples, ages 20–66, are displayed. Tested samples were from Caucasian and African-American donors who did not have multiple sclerosis. Specific IgE/(kappa + lambda)-positive results are confined to single, non-dimer participating epitope.

**Table 1:** Illustrated is the method employed in estimating the average diameter, in Ångströms, of the twenty standard amino acids. The method entails (1) estimating the nanometers diameter of each non-alanine amino acid relative to the known diameter of alanine, 0.69 nanometer, using the formula, amino acid molar mass/alanine molar mass ×0.69 nanometer(s); (2) multiplying each estimated amino acid diameter times 10 in order to convert each amino acid diameter from nanometers to Ångströms; (3) summing the Ångströms diameters and dividing by 20 to yield an average amino acid diameter per amino acid equal to 10.6 Ångströms.

Estimation of average amino acid diameter				
Molar mass		Amino acids	Nanometer diameter	Diameter in Ångströms
89.1	A	Alanine	0.6**	6.9
132.1	N	Asparagine	1.02	10.2
133.1	D	Aspartic acid	1.03	10.3
121.6	C	Cysteine	0.94	9.4
147.1	E	Glutamic acid	1.14	11.4
146.1	Q	Glutamine	1.13	11.3
75.1	G	Glycine	0.58	5.8
115.1	P	Proline	0.89	8.9
105.1	S	Serine	0.81	8.1
181.2	Y	Tyrosine	1.40	14.0
174.2	R	Arginine	1.35	13.5
155.2	H	Histidine	1.20	12.0
131.2	I	Isoleucine	1.02	10.2
131.2	L	Leucine	1.02	10.2
146.2	K	Lysine	1.13	11.3
149.2	M	Methionine	1.16	11.6
165.2	F	Phenylalanine	1.28	12.8
119.1	T	Threonine	0.92	9.2
204.2	W	Tryptophan	1.58	15.8
117.5	V	Valine	0.91	9.1

Average Ångströms diameter per amino acid →				**10.6**

**[14].

**Table 2:** Concentrations of mimotopic peptide constructs used in application to individual microplate test wells are listed alongside each construct sequence. The basic procedure is described in Section 2.

Microgram of mimotopic peptide construct per milliliter of coating buffer for MS test microplate wells		
Peptide amino Acid Sequence of amino-ADOOA-ADOOA-peptide Construct ADOOA-peptide construct	Peptide Construct *μ*g/mL Concentration	Construct molar mass (kilodaltons)
(1) ADARM	0.26	0.56
(2) HSYQE	0.31	0.66
(3) RNVRF	0.33	0.69
(4) VTLRI	0.28	0.6
(5) IENLH	0.29	0.62
(6) NLHRTFFE	0.5	1.06
(7) KTGQF	0.27	0.58
(8) DNTFKD	0.39	0.82
(9) HGRTQ	0.28	0.6
(10) LQTIQE	0.34	0.73
(11) PKNAW	0.34	0.73
(12) QDTAVT	0.3	0.63
(13) YKDSHHPA	0.45	0.95
(14) DNEVFGEA	0.5	0.9

**Table 3:** Psychotropic pharmaceuticals and other agents shown to be immunosuppressive are listed (left column) alongside their specific suppressive effects (middle column) and describing literary citations (right column). Specific citations are listed in the References section.

Anti-inflammatory Agent (ID no. marked yellow on individual plots)	Immunosuppressive effect	References
(1) Mesalazine	Potent and specific inhibitor of nuclear factor kappa B.	[24]
*Anticonvulsants*		
(2) Dilantin (phenytoin sodium)	Humoral immune suppressant.	[25]
(3) Zonisamide	Suppression of IFN-gamma Production by Lymphocytes.	[26]
*Atypical Antipsychotics*		
(4) Olanzapine (Zyprexa, etc.)	Suppress tumor necrosis factor, (TNF)-alpha, interleukin (IL)-6, and upregulates IL-10.	[27]
*Benzodiazepines*:		
(5) Alprazolam ( Xanax)	Inhibits proliferative responses of both B- and T-cells.	[28]
(6) Clonazepam	Depression of cellular and humoral immune response.	[29]
(7) Diltiazem	Induces direct immunosuppression.	[30].
(8) Diazepam (Valium)	Markedly suppresses Antigen-specific antibody production and T-cell reactivity.	[31]
*Colesterol Lowering Drugs*		
(9) Atorvastatin (Lipitor)	Increases in IL-10 production. IL-10 mediates immune suppression.	[32]
(10) Fenofibrate (reduces lipoproteins)	A Peroxisome proliferator-activated receptor alpha agonist.	[33]
(11) Pravastatin	B, lymphocyte and T lymphocyte suppression.	[34]
(12) Rosuvastatin (Crestor)	Posttranscriptional level of genetic expression of inflammatory process.	[35]
(13) Simvastatin (Zocor)	Mediates induction of Foxp3(+) T Cells Which Mediate Immuno-Suppression.	[36]
*Dopamine Reuptake Inhibitors *(*Antidepressants*)		
(14) Bupropion (Wellbutrin, etc.)	Involved in inhibiting neuroimmunomodulation.	[37]
*Serotonin*—*Norepinephrine Reuptake Inhibitors *(*SNRIs, Antidepressants*):		
(15) Venlafaxine	Suppresses proinflammatory cytokines.	[38]
*Selective Serotonin Reuptake Inhibitors (SSRIs, antidepressants):*		
(16) Paroxetine (trade names: Seroxat, Paxil)	Inhibits splenocyte viability.	[39]
	Decreases CD4 T-helper cells.	[40]
(17) Fluoxetine (Prozac)	Decreases T Lymphocyte Activity.	[41]
(18) Sertraline hydrochloride (Zoloft)	Suppression of antigen-specific T(H)1 responses. Inhibition of interferon gamma and stimulation of interleukin-10.	[42]
(19) Clomipramine	As per sertraline.	[43]
(20) Trazodone (Desryl, Oleptro, Beneficat, Deprax, Desirel, Molipaxin, Thombran, Trittico, Mesyrel).	As per sertaline.	[43]
*Other Immunnosuppressants* (1):		
(21) Amantadine	Inhibits antigen-specific T- and NK-Cell Responses.	[44]
(22) Amitriptyline (Elavil, Tryptizol, Laroxyl, Sarotex)	Decrease in the Proliferation of Slenocytes and in NK Activity.	[45]
(23) Clonidine (a direct-acting *α*2 adrenergic agonist).	Stimulates production of IL-10 (an anti-Inflammatory cytokine that reduces serum antibody production.)	[46]
(24) Depakote (Valproate semisodium used to treat major depressive disorder.)	Suppresses IL-6- and/or IL-6R-related mechanisms.	[42]
(25) Donepezil (Aricept)	Reversible Acetylcholinesterase Inhibitor. Suppresses Neuroinflammation of the Brain.	[47]
(26) Mitoxantrone (Novantrone)	Chemotherapeutic agent, depletes B cells.	[48]
(27) Levoxyl (Levothyroxine, Synthroid).	Inhibits cytokine production in T cells.	[49]
(28) Warfarin (Coumadin)	Suppresses IL-6 secretion. Serves as immunosuppressant.	[50]
*Other Immunnosuppressants* (2):		
(29) Heroin and Methadone.	Suppression of Cellular and Humoral Immunity.	[51]
(30) Morphine.	Suppression of Cellular and Humoral Immunity.	[51]
(31) Oxycodone & Propoxyphene	Suppression of Cellular and Humoral Immunity.	[51]
(32) Prednisone	Catabolic Steroid. Suppression of Cellular and Humoral Immunity.	

**Table 4:** Listed are structurally unique, mimotopic, peptides serving as diagnostic and therapeutic antigens. Respective peptide hydrophilic indices (HIs) are displayed in columns 5 and 7. Peptides used for initial diagnosis can be of maximum, unique length (column 4) or fractionated into pentameric (single epitope) equivalents (column 6). Either takes place. Each test peptide is synthesized with an amino-ADOOA-ADOOA linker. The amide group is used for covalent coupling to microplate wells, and the ADOO A-ADOOA construct, being very hydrophilic, solubilizes all peptides, especially those that are hydrophobic. The selected myelin dimer-cornerstone, peptide homologues for therapy are therapeutically promising because of their in vivo (a) net hydrophilicity for good solubility; (b) relatively small size for intravascular permeation; and (c) ability to simultaneously abrogate formation of up to 20 pathological myelin dimers by administering just 4 pentameric plus 2 hexameric peptides (bold highlighted in columns 4 and 6) in lieu of needing to employ up to 28 individual therapeutic pentamers and having to confront individual solubility issues, and so forth.

	MS-specific peptides sequences for diagnosis and therapy					
Myelin protein			Full-length mimotopic peptides	HI	Constituent, single-epitope, pentameric equivalents	HI
PLP	m.s.		**ADARM**	3.7	**ADARM**	**3.7**
MOG	m.s.		**HSYQE**	0.7	**HSYQE**	**0.7**
			RNVRF	2.2	RNVRF	2.2
			VTLRI	−2.5	VTLRI	−2.5
MOG	i.c.		IENLH	−0.9	IENLH	−0.9
			NLHRTFE	1	NLHRT	0.5
					LHRTF	−2.2
					HRTFE	2.6
			**KTGQF**	0.3	**KTGQF**	**0.3**
MBP	i.c.	Isoforms 1,2	DNEVFGEA	4.7	DNEVF	2.2
		"			NEVFG	2.0
		"			EVFGE	2.0
		"			VFGEA	−1.5
		Isoforms 1,2	**QDTAVT**	0.4	**QDTAV**	**0.8**
		"			**DTAVT**	**0.2**
		Isoforms 1,2	PKNAW	−1	PKNAW	−1
MBP	i.c.	Isoforms 1,2	**DNTFKD**	6.3	**DNTFK**	**3.3**
		"			**NTFKD**	**3.3**
		Isoforms 1,2	LQTIQE	−1	LQTIQ	-3.6
		"			QTIQE	1.2
MBP	i.c.	Isoform 1	KDSHHPA	3	KDSHH	5.3
		"			DSHHP	2.3
		"			SHHPA	−1.2
		Isoforms 1,3	**HGRTQ**	2.3	**HGRTQ**	**2.3**
MBP	i.c.	Isoforms 3	YKDSHHPA	2.5	YKDSH	2.5
					KDSHH	5.3
					DSHHP	2.3
					SHHPA	−1.2

**Bold**: cornerstone epitopes.
